# Urokinase Gene 3′-UTR T/C Polymorphism Is Associated with Malignancy and ESRD in Idiopathic Membranous Nephropathy

**DOI:** 10.1155/2014/425095

**Published:** 2014-04-14

**Authors:** Cheng-Hsu Chen, Shih-Yin Chen, Kuo-Hsiung Shu, Mei-Chin Wen, Chi-Hung Cheng, Ming-Ju Wu, Tung-Min Yu, Ya-Wen Chuang, Fuu-Jen Tsai

**Affiliations:** ^1^Division of Nephrology, Department of Internal Medicine, Taichung Veterans General Hospital, Taichung, Taiwan; ^2^Department of Internal Medicine, Chiayi Branch, Taichung Veterans General Hospital, Chiayi, Taiwan; ^3^School of Medicine, China Medical University, Taichung, Taiwan; ^4^Department of Life Science, Tunghai University, Taichung, Taiwan; ^5^Graduate Institute of Chinese Medical Science, China Medical University, Taichung, Taiwan; ^6^Department of Pathology, Taichung Veterans General Hospital, Taichung, Taiwan; ^7^Department of Medical Genetics, China Medical University Hospital, No. 2 Yuh Der Road, Taichung 404, Taiwan

## Abstract

Idiopathic membranous nephropathy (MN) is one of the most common causes of nephrotic syndrome in adults, and 25% of MN patients proceed to ESRD. Urokinase plasminogen activator (uPA) may play an important role in reducing renal fibrosis. This study was conducted to clarify the relationship between uPA gene polymorphisms and clinical manifestations of MN. We recruited 91 biopsy-diagnosed MN patients and 105 healthy subjects. Genotyping of uPA gene 3′-UTR T/C polymorphism was performed by polymerase chain reaction methods. The genotype distribution had no effect on the development of MN. Thirteen patients (15.9%; *P* = 0.008) acquired malignancies and seventeen (20.7%; *P* = 0.006) patients progressed to ESRD with the C/C genotype, but no patients with the T/C genotype did. In conclusion, we demonstrated that the presence of the uPA gene 3′-UTR C/C genotype was associated with ESRD as well as acquired malignancies in MN patients. These findings should prompt specific considerations for the treatment of MN patients to maintain a balance between treating disease entities and protecting the immune system from cancers.

## 1. Introduction

End-stage renal disease (ESRD) is a major public health problem in Taiwan, which had the highest incidence and prevalence country in the world in 2007: 415 and 2,288 per one million people, respectively [1]. The annual mortality rate was 7.55 in 2007 [2] and the national prevalence of chronic kidney disease was 11.93% [3]. Renal disease was listed as the tenth leading cause of death at 13.2/10^5^ population in the 2009 Annual Report of the U.S. National Institutes of Health and the National Institute of Diabetes and Digestive and Kidney Diseases [1].

Membranous nephropathy (MN) is one of the common causes of ESRD in adult glomerulonephritis, also known to be the second most frequent cause of primary glomerulonephritis in Chinese people [4]. It is a prototype of an immune-mediated glomerular disease and is characterized by abundant, nonselective proteinuria and variable clinical course and prognosis [5–7]. The renal function and the course in MN are more strongly correlated with the degree of tubulointerstitial damage than with the extent of the glomerular lesion [8, 9], but the pathogenesis of the interstitial inflammation and fibrosis is unclear.

Genetic and environmental factors may contribute to disease progression and renal fibrosis in most types of renal disease. Identifying the genetic mechanism which may be related to the high incidence of MN is crucial for improving the current situation in Taiwan. In our previous study, we demonstrated that the genotype distribution of plasminogen activator inhibitor type 1 (PAI-1) had no effect on the development of MN, and the 4G/4G genotype had significantly poorer creatinine clearance than the 4G/5G or 5G/5G genotypes in MN patients [10]. The frequency distribution of the G allele in genotype polymorphism of rs437168 (exon17) in the NPHS1 gene was significantly higher in MN patients than in controls, and a stratified analysis revealed a high disease progression in the AA genotype of rs401824 (5′UTR) and GG genotype of rs437168 (exon17) patients who were associated with a low rate of remission [11].

Urokinase plasminogen activator (uPA), a plasminogen activator synthesized by the kidney and other cells that triggers the cleavage of plasminogen to plasmin and hence induces fibrinolysis, may play an important role in reducing renal fibrinosis [12, 13]. Urinary uPA was significantly lower in patients with intraglomerular fibrin deposition than in patients without fibrin deposition. The decrease of urinary uPA levels and diminution of isolated intraglomerular plasminogen activator activity contribute to the progression of primary glomerular diseases [14]. UTRs are known to play crucial roles in the posttranscriptional regulation of gene expression, including modulation of the transport of mRNAs out of the nucleus and of translation efficiency, subcellular localization, and stability [15–17]. The urokinase gene 3′-UTR “T” allele is highly associated with calcium stone disease [18] and oral cancer [19]. The present study was therefore conducted to investigate the frequency distribution of urokinase gene 3′-UTR gene polymorphism associated with the risk of MN patients and healthy individuals in Taiwan and to explore the correlation of the clinical manifestation in different gene polymorphisms.

## 2. Methods

### 2.1. Study Population

We recruited a cohort of 92 biopsy-diagnosed MN patients and 105 healthy subjects, who served as controls during January 2000–December 2002. The follow-up period of MN patients was from their renal biopsy date to June 2011. Patients who had secondary MN with malignancy, chronic infectious diseases (including infections with hepatitis B and hepatitis C viruses), lupus nephritis, or drug-induced diseases were excluded from this study. The patient characteristics and medical records were reviewed, including demographic variables, clinical and laboratory data for the disease courses, vascular events, and treatment regimens as well as their responses. Informed consent was obtained from all participating individuals and the study protocol complied with the ethical guidelines of our hospital (VGHTC IRB number C08159).

The selection of treatment modality, either supportive or aggressive with immunosuppressants, was based on the treating physician's decision. The supportive therapy usually included diuretics, angiotensin converting enzyme inhibitors (ACEIs), and/or angiotensin II receptor blockers (ARBs), depending on the patient's symptoms. The immunosuppressive therapies included any of the following regimens: (1) prednisolone 1 mg/kg/day alone, (2) a six-month course of corticosteroids alternating with chlorambucil at a dose of 0.2 mg/kg/day every other month [20] or cyclophosphamide 1.5–2.0 mg/kg/day, or (3) cyclosporine A (CyA, Novartis Inc., Basel, Switzerland) 3–5 mg/kg/day with or without prednisolone.

### 2.2. Responses and Outcomes

The responses to therapy were defined as follows: (1) no response, (2) partial remission: a proteinuria reduction of more than 50% or final proteinuria between 0.2 and 2.0 g/day, and (3) complete remission: proteinuria less than 0.2 g/day. The progression of renal disease was defined as doubling baseline serum creatinine (Cr) values or ESRD. ESRD was defined as required renal replacement therapy.

### 2.3. Renal Biopsy Review

The histological staging was based on histological lesion, including glomerular lesion [16], tubulointerstitial lesion, focal glomerulosclerosis [17], and fibrointimal lesion. The renal biopsy specimens were reviewed by a nephropathologist, who was blinded to patients' clinical history, renal function, and urokinase gene 3′-UTR T/C polymorphism. A semiquantitative scoring system was adopted using a scale of 0 (none), 1 (mild: less than 25%), 2 (moderate: 25 to 50%), and 3 (severe: more than 50%) for the assessment of tubulointerstitial change and glomerular sclerosis/obsolescence under light microscopy. Staging of disease was also determined according to findings using electron microscopy.

### 2.4. Determination of Urokinase Gene 3′-UTR T/C Polymorphism

PCRs were carried out to a total volume of 50 *μ*L containing genomic DNA, 2 to 6 pmol of each primer, 1X Taq polymerase buffer (1.5 mM MgCl_2_), and 0.25 U AmpliTaq DNA polymerase (Perkin Elmer, Foster City, CA). The primer for the urokinase gene 4065 T/C polymorphism was designed as 5′-CCGCAGTCACACCA AGGAAGAG-3′ and 5′-GCCTGAGGGTAAAGCTA TTGTCGTGCAC-3′, according to the published data from Medline (STS Accession number G27040). PCR amplification was performed in a programmable thermal cycler GeneAmp PCR System 2400 (Perkin Elmer). The cycling condition for urokinase gene 3′-UTR T/C polymorphism was set as follows: one cycle at 94°C for 5 minutes, 35 cycles at 94°C for 30 seconds, 58°C for 30 seconds, and 72°C for 40 seconds, and one final cycle of extension at 72°C for 7 minutes.

The PCR product of 210-bp was mixed with 2 U* ApaL* I (New England Biolabs, Beverly, MA) and the reaction buffer, according to the manufacturer's instructions. The restriction site was designed to be located at the allele of 3′-UTR (T) to form a digestible site. Two fragments of 185-bp and 25-bp will be present if the product is digestible. The reaction was incubated for 3 hours at 37°C. Then, 10 *μ*L of the product was loaded into 3% agarose gel plates containing ethidium bromide for electrophoresis. The polymorphism 343cbe was divided into three groups: digestible (T/T homozygote), indigestible (C/C homozygote), and C/T heterozygote.

### 2.5. Urine uPA Measurement

A 20 mL of urine was collected into clean 15 mL tubes for immediate freezing and storage in −20°C freezers. Urine uPA levels were determined by using the suPARnostic ELISA kit (ViroGates, Copenhagen, Denmark). The assay comprised plates precoated with a catching monoclonal antibody for loading the sample and an HRP-labelled detection monoclonal antibody that was added to the sample dilution buffer. Briefly, 25 *μ*L of urine sample was mixed with 225 *μ*L of dilution buffer added to the plates and incubated for one hour. After washing the plates, 50 *μ*L of substrate was added for 20 min and the reaction was stopped with 50 *μ*L 0.5 M H_2_SO_4_. Plates were measured at 450 nm in a spectrophotometer.

### 2.6. Statistical Analysis

Continuous variables are expressed as mean and standard deviation. Urokinase gene 3′-UTR T/C genotype and allele frequencies between MN and normal controls were compared using Chi-square analysis. The relationships between urokinase gene 3′-UTR T/C genotypes, patient characteristics, and histology were also compared by Chi-square test. Differences in various clinical parameters among T/T, T/C, and C/C genotypes were compared using the analysis of variance (ANOVA).

Kaplan-Meier survival analysis was used to determine kidney survival and patient survival. The survival rate among different urokinase gene 3′-UTR T/C genotypes was compared by means of a two-sided Log-rank test. Differences were considered statistically significant when *P* < 0.05. All analyses were performed using SPSS 10.0 (SPSS Inc., Chicago, IL).

## 3. Results

### 3.1. Population Study

The distribution of urokinase gene 3′-UTR (SNP4065) polymorphism among MN and healthy control subjects was not significantly different, as shown in Table 1. Neither allelic frequency nor carriage rates of urokinase gene 3′-UTR polymorphism were observed in MN or healthy control subjects (Table 1). No MN patients carried the T/T homozygote. Only 9 (13%) MN patients carried the T/C genotype, whereas 82 (90%) MN patients carried the C/C genotype, which was similar to the percentage for healthy controls (Table 1).

### 3.2. Relationship between Urokinase Gene 3′-UTR T/C Polymorphisms and Clinical Features of MN

The minimal follow-up duration was 1 year, except for four patients who died of pneumonia; two had respiratory failure (0.9 months and 1.4 months), one had septic shock (2.2 months), and one had subdural hemorrhage with urosepsis (1.5 months), respectively. The clinical features of the 2 genotypes of urokinase gene 3′-UTR polymorphism are shown in Table 2. There were no differences in gender distribution, age at onset, duration of follow-up, body mass index, mean blood pressure (MBP), hematuria, or proteinuria. The baseline laboratory data revealed similarities between the serum creatinine level (Cr), creatinine clearance (CCr) level, and daily urinary protein excretion (DUP) in Table 2. After a mean duration of 9.5 ± 6.0 years' follow-up, the last Cr measurement in patients with the C/C genotype (3.3 ± 4.3 mg/dL) was higher than that in patients with the T/C genotype (1.2 ± 0.4 mg/dL), but the difference did not reach statistical significance. The last CCr measurement (52.1 ± 39.2 mL/min) in patients with the C/C genotype was lower than that in patients with the T/C genotype (76.4 ± 26.7 mL/min), though without statistically significance (*P* = 0.075) in Table 2. The pathological features also disclosed no difference between the MN grade, percentage of glomerulosclerosis, tubulointerstitial fibrosis score, or fibrointimal atherosclerosis score between the two genotypes in MN patients (data not shown).

### 3.3. Relationship between Urokinase Gene 3′-UTR T/C Polymorphisms and Outcomes in MN

There is no consensus on the standard treatment for patients with idiopathic membranous nephropathy. Generally, high-risk patients are treated with immunosuppressive therapy, such as steroids in combination with chlorambucil or cyclophosphamide, and cyclosporine. Although our patients received the best available treatment regimens, only 42 (51.2%) patients with the C/C genotype and 6 (66.7%) patients with the T/C genotype achieved complete remission. Forty-seven (57.3%) patients with the C/C genotype and 3 (33.3%) patients with the T/C genotype had disease progression. MN progressed to ESRD in 17 (20.7%) patients with the C/C genotype, but no MN patients with the T/C genotype had progression to ESRD (*P* = 0.006, Table 3). These results indicated that MN patients who carry the C/C genotype in the 3′-UTR urokinase gene have poor response to treatment modalities and aggravated renal function leading to ESRD. The urine uPA levels were compared to strengthen the functional study of uPA genes 3′-UTR C/C (*n* = 22) and T/C (*n* = 6) genotypes, without significant difference between two genotypes in Table 2.

The cardiovascular events were similar in both genotypes. Thirteen (15.9%) patients with the C/C genotype developed malignancies during follow-up, whereas no patients with the T/C genotype did (*P* = 0.008, Table 3). Most of the patients received immunosuppressive therapies, including prednisolone (12), a combination of prednisolone and cytotoxic agents (5) and CsA (3) for MN, and one received ACEI therapy only. The median period of onset of malignant neoplasms was 9.9 ± 6.1 years. The pattern of malignancy (Table 4) was different from that of the three leading types of cancer in Taiwan: lung cancer, hepatoma, and breast cancer. The MN patients with malignancy received surgical intervention in 8 (61%) and chemotherapy in 3 (23%). Five of them were mortality with function kidney, and four patients renal failure before death.

### 3.4. Survival Analysis of MN Patients with Different 3′-UTR Genotypes of the Urokinase Gene

**Figure 1:**
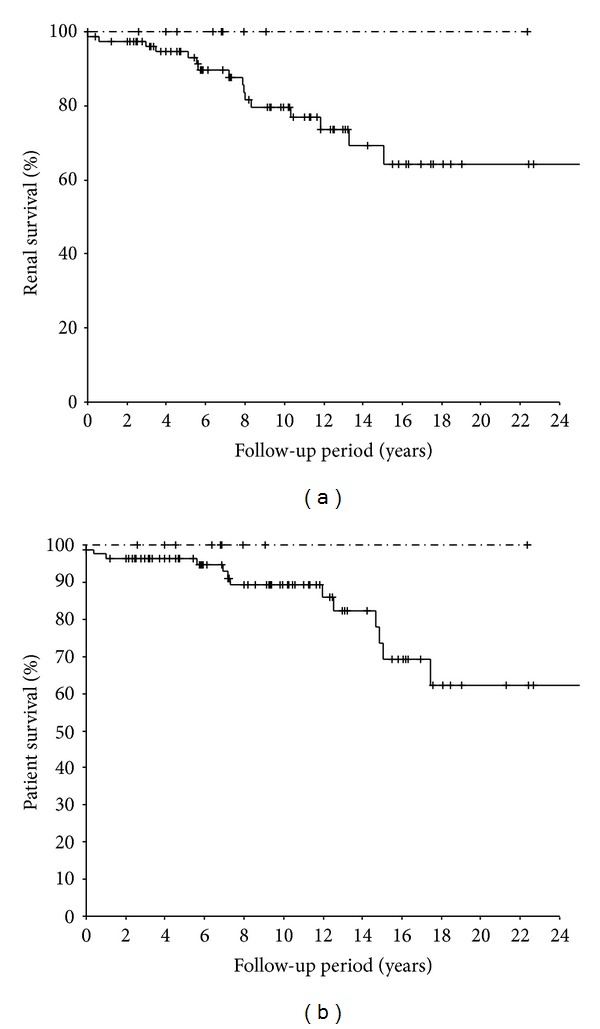
(a) Kaplan-Meier plot of renal survival stratified by urokinase gene 3′-UTR gene polymorphism (C/C: — T/C: -·-) in membranous nephropathy. Mean renal survival was 19.8, 95% confidence interval 17.1–22.4. (b) Kaplan-Meier plot of patient survival stratified by urokinase gene 3′-UTR gene polymorphism (C/C: — T/C: -·-) in membranous nephropathy. Mean survival was 20.6 yrs. 95% confidence interval: 18.2–23.1 in C/C group.

Figure 1 shows the Kaplan-Meier curves for renal survival and patient survival according to the distributions of the 3′-UTR genotypes of the urokinase gene. Although there was a trend towards better renal survival and patient survival in T/C patients (100%), this difference was not significant due to the small sample size.

## 4. Discussion

The current study showed that urokinase gene 3′-UTR polymorphism was not correlated with the development of MN. However, we clearly demonstrated that the presence of the C/C allele in MN patients was associated with ESRD and possibly with the occurrence of malignant neoplasms. These data strongly suggest poor prognosis for MN patients with the C/C genotype. Although renal survival and patient survival rates were not significantly different, a trend towards improved survival was found in patients with the T/C genotype. Because MN is an insidious disease with a protracted clinical course, the follow-up duration of the current study may be too short and the sample size may be too small to detect a meaningful difference in survival between the two subgroups.

The exact mechanism by which the C/C genotype exerts its detrimental effect is not fully understood. Our previous study demonstrated that the presence of the 4G allele was associated with renal deterioration in MN patients [9]. The expression of PAI-1, an inhibitor of uPA and tissue plasminogen activator (tPA), in injured kidney is associated with enhanced recruitment of interstitial macrophages and myofibroblasts not only from increased matrix protein synthesis but also from decreased degradation by connective tissue proteases [21, 22]. uPA, which is copiously produced by proximal and distal tubules, is a logical source of endogenous renal antifibrotic activity. However, uPA is normally excreted apically into the urinary space, and whether significant interstitial delivery occurs when the kidney is damaged is unknown [22]. In a primary glomerulopathy study, the urinary uPA levels were significantly reduced by intraglomerular fibrin deposition, which suggests that a decrease in urinary uPA levels and diminution of isolated intraglomerular plasminogen activator activity contribute to disease progression [14].

In this study, we demonstrated that MN patients with the C/C genotype in the 3′-UTR urokinase gene had poor response to treatment modalities and aggravated renal function leading to ESRD. The interaction of RNA-binding proteins with 5′- or 3′-untranslated regions (UTRs) of mRNA is the translational control mechanism. Protein-mediated interactions between transcript termini result in the formation of an RNA loop in all species, which is thought to increase translational efficiency and to permit regulation by new mechanisms, particularly 3′-UTR-mediated translational control [15–17, 23]. In many of the previous studies, the urokinase gene 3′-UTR T allele was associated with higher incidence of calcium oxalate stone disease [18], rheumatoid arthritis [24], oral cancer [19], prostate cancer [25], bladder cancer [26], and Alzheimer's disease [27], but some other studies indicated that the T allele is not associated with calcium oxalate nephrolithiasis [28] or bronchopulmonary dysplasia in ventilated preterm infants [29]. To our knowledge, there was no correlation of uPA activity with urokinase gene 3′-UTR genotypes in previous studies, and we did not find such correlation in this study. It was recently reported that uPA activity may have organ-specific effects during fibrotic response, and various experimental interventions with high uPA could decrease fibrosis in the lungs and liver and increase fibrosis in the heart in mice [30, 31]. In a mouse UUO model, there was no significant difference in the degree of renal fibrosis between uPA wild-type and knockout mice [32], but uPA may interact with several distinct cellular receptors, including some that promote and others that inhibit renal fibrosis [30, 33]. The most important cellular receptor is the high-affinity receptor for urokinase (uPAR). During renal insult, the inducible uPAR enhanced expression of some renal tubules, inflammatory cells, and interstitial myofibroblasts [34]. Studies on uPAR genetically deficient mice have demonstrated that uPAR plays a protective role during the kidney's response to injury [34–36]. The mechanism of renal fibrosis is complex and not yet clear in humans. Therefore, further investigation of the relationship between uPA activity and uPAR in serum and pathology is needed.

In this study, 13 MN patients (15.9%) with the C/C genotype acquired malignancies during long-term follow-up. uPA has a proteolytic effect on degradation of the extracellular matrix (ECM), which allows malignant cells to invade locally and eventually spread distally. High levels of uPA components have been shown to predict adverse outcome in different types of malignancy and are consistent with cancer progression [36]. The present study demonstrated that a high rate of malignancy was associated with the urokinase gene 3′-UTR C/C genotype in MN, but the relationship needs further clarification before definitive conclusions can be made. Our 13 MN patients received strong immunosuppressive agents for several years to counteract the poor response of proteinuria; thus, most of their cancers were those associated with known or suspected viral causes, such as lymphoma, cervical cancer, and skin cancer [37]. The results were similar to the results for organ transplant recipients, which suggest that immunity was oversuppressed in our strategy to manage resistant MN. Patients with malignancies are frequently exposed to risk for renal injuries associated with disease-related or iatrogenic causes. Nephrotoxicity is a potential adverse effect of anticancer agents (e.g., gemcitabine and cisplatin) especially in patients with vulnerable chronic kidney disease (CKD) like MN. Increased understanding of the mechanism of renal injury by these agents, it is important to avoid adverse effects on CKD patients by adjusting their dosage [36, 37].

Although our study had the limitation of small sample size, there were no significant differences among the initial clinical characteristics and pathological features, the modes of treatment, or the follow-up duration in the two genotypes. On the other hand, the urokinase gene 3′-UTR T allele is rare in Taiwan [13, 14]. The small sample population and rare allele frequency might have a reduced chance of detecting a true effect and also could reduce the statistically significant result that reflects a true effect.

In conclusion, we demonstrated that the presence of the urokinase gene 3′-UTR C/C genotype was associated with ESRD as well as acquired malignancies in MN patients. These findings should prompt specific considerations for the treatment of MN patients to maintain a balance between treating disease entities and protecting the immune system from cancers.

## Figures and Tables

**Table 1:** Distribution of 3′-UTR gene polymorphism (SNP4065) of urokinase gene among idiopathic membranous nephropathy and healthy control subjects.

Genotype	MN (*N* = 91)	Control (*N* = 105)	*P* value
Male gender	50 (54)	61 (58)	0.325^a^
Age (yrs)	57.8 ± 16.4	49.8 ± 14.4	0.000^b^
Genotype of urokinase gene			
C/C	82 (90%)	90 (86%)	0.237^c^
C/T	9 (10%)	15 (14%)	
T/T	0 (0%)	0 (0%)	
Allelic frequency			
C	173 (95)	195 (93)	0.238^a^
T	9 (5)	15 (7)	
Allelic carriage			
C	91 (100)	105 (100)	0.318^a^
T	9 (10)	15 (14)	

Number in the parenthesis is percentage.

^a^Yates' correction of contingency.

^b^Mann–Whitney *U* test.

^c^Chi-square test.

Patient age, which was used as a covariate to adjust the genotype in the study population by multinomial logistic regression, as not significantly different between control and MN groups.

**Table 2:** The clinical characteristics and urokinase gene 3′-UTR gene polymorphism and its activity.

	C/C (*n* = 82)	C/T (*n* = 9)	*P* value
Male gender (%)	42 (51.8%)	7 (77.8%)	0.121
Age of onset (yrs)	52.5 ± 15.7	51.4 ± 25.2	0.847
Age of biopsy (yrs)	58.2 ± 15.4	53.9 ± 25.0	0.463
Follow-up period (yrs)	9.6 ± 6.0	7.8 ± 5.8	0.390
BMI (Kg/M^2^)	24.7 ± 3.6	24.0 ± 3.3	0.557
MBP (mmHg)	98.6 ± 12.2	101.5 ± 24.2	0.553
Albumin (mg/dL)	2.5 ± 0.6	2.7 ± 0.5	0.338
Cholesterol (mg/dL)	347.7 ± 137.3	298.9 ± 113.3	0.307
Triglyceride (mg/dL)	236.3 ± 165.0	161.2 ± 119.5	0.189
Cr_initial_ (mg/dL)	1.3 ± 1.1	1.4 ± 1.1	0.788
DUP_initial_ (g/day)	7.0 ± 8.2	7.1 ± 4.7	0.974
CCr_initial_ (mL/min)	87.6 ± 41.2	81.0 ± 45.6	0.655
PT (second)	11.2 ± 0.8	11.6 ± 0.8	0.290
aPTT (second)	27.0 ± 5.2	30.0 ± 3.2	0.144
Cr_final _(mg/dL)	3.3 ± 4.3	1.2 ± 0.4	0.150
DUP_final_ (g/day)	2.6 ± 3.5	1.1 ± 1.3	0.197
CCr_final_ (mL/min)	52.1 ± 39.2	76.4 ± 26.7	0.075
Proteinuria ≧ 3.5 g/day	28 (34.1%)	3 (33.3%)	0.838
Hematuria	46 (56.1%)	4 (44.4%)	0.374
Lower leg edema	69 (84.1%)	7 (77.8%)	0.451
Urine uPA (ng/mL)	6.17 ± 4.68	6.06 ± 5.37	0.962

BMI: body mass index; MBP: mean blood pressure; DUP: daily urinary protein excretion; CCr: creatinine clearance. All data are presented as mean ± SD; urine uPA: urine urokinase plasminogen activator. There were only 28 urine samples collected from patients with C/C (*n* = 22) and C/T genotypes (*n* = 6) of urokinase gene 3′-UTR gene polymorphism for the functional study of urine uPA.

**Table 3:** 3′-UTR of urokinase gene polymorphism and clinical outcome.

	C/C (*n* = 82)	C/T (*n* = 9)	*P* value
Cardiovascular events	17 (20.7)	2 (22.2)	0.917
Malignancy	13 (15.9)	0 (0.0)	0.008*
Complete remission	42 (51.2)	6 (66.7)	0.641
Disease progression	47 (57.3)	3 (33.3)	0.154
ESRD	17 (20.7)	0 (0.0)	0.006*

Distribution was analyzed by Chi-square test.

*Measured by Kendall's Tau-b significant *P* value < 0.05.

**Table 4:** The malignant neoplasm in C/C genotype of 3′-UTR of urokinase gene polymorphism in MN patients and their presentation.

Malignancy	Malignancy duration (yrs)			Management
	Diagnosis	Renal failure	Mortality	
Lymphoma (3)				
Diffuse large B cell lymphoma, brain	25.0	Before	2.6/death	Chemotherapy
Malignant lymphoma, mixed large and small cleaved cell, B phenotype, with bone marrow involvement, stage IV	5.5	1.7/function	1.7/death	Chemotherapy
Angioimmunoblastic T-cell lymphoma	5.5	0.1/function	0.1/death	No
Skin cancer (2)				
Basal cell carcinoma, left nasal base and medial canthus	15.5	1.7/function	1.7/death	Incision
Squamous cell carcinoma, skull	2.9	2.2/failure	3/death	Incision
GI tract malignancy (3)				
Adenocarcinoma, stomach	15.0	0.1/function	0.1/death	No
Adenocarcinoma, stomach	7.6	2.5/function	2.5/survival	Subtotal gastrectomy
Adenocarcinoma, rectum	6.2	7.3/function	7.3/survival	polypectomy
GU tract malignancy (2)				
Prostate adenocarcinoma	9.5	Before	5.9/death	Radical prostatectomy
Renal cell carcinoma, left kidney	3.2	4.3/failure	5.2/death	Left nephrectomy
Gynecology malignancy (2)				
Squamous cell carcinoma, cervix	10.9	1.2/function	1.2/survival	Vaginal hysterectomy
Squamous cell carcinoma, cervix	10.6	Before	2.3/survival	Vaginal hysterectomy
Respiratory tract (1)				
Adenocarcinoma, LLL, lung	11.9	0.6/function	0.6/death	Chemotherapy

Before: renal failure before diagnosis of malignancy.
